# First-Line Nivolumab Plus Ipilimumab in Inoperable Epithelioid Malignant Pleural Mesothelioma: Real-World Experience From an Indian Tertiary Care Center

**DOI:** 10.7759/cureus.113313

**Published:** 2026-07-24

**Authors:** Avi Shah, Ankit Agarwal, Vishva Rajpuria, Shiv Ghodasara, Jagdish Swami, Vibhav Kanth, Hiren Dangar, Ashish Jakhetiya

**Affiliations:** 1 Medical Oncology, Geetanjali Medical College, Udaipur, IND; 2 General Medicine, Smt. Nathiba Hargovandas Lakhmichand (NHL) Municipal Medical College, Ahmedabad, IND; 3 Surgical Oncology, Geetanjali Medical College, Udaipur, IND

**Keywords:** dual immune checkpoint inhibitors, malignant pleural mesothelioma (mpm), real world data, safety and efficacy, unresectable pleural mesothelioma

## Abstract

Background

Malignant pleural mesothelioma (MPM) is a highly aggressive, rare malignancy with poor prognosis. Standard frontline treatments have traditionally relied on platinum-based chemotherapy doublets, which offer limited long-term survival benefit. This study evaluates the efficacy, clinical-demographic profile, and safety of first-line dual checkpoint inhibition using nivolumab and ipilimumab in patients with inoperable MPM within an Indian tertiary care setting.

Materials and methods

A retrospective study was conducted from January 2021 to August 2023 at a single tertiary care center in India. Fifteen patients aged ≥ 18 years with histologically confirmed, previously untreated, metastatic/inoperable epithelioid MPM and an Eastern Cooperative Oncology Group (ECOG) performance status (PS) of 0-2 were included. Patients received nivolumab (240 mg intravenously every two weeks) combined with ipilimumab (1 mg/kg intravenously every six weeks) until disease progression or unacceptable toxicity. Response assessments were performed using positron emission tomography (PET) scans at three months using modified RECIST criteria. The primary objective of this study was to evaluate the real-world overall survival (OS), including median OS and one-year OS and safety profile. Secondary objectives included the assessment of objective response rate (ORR) at first reassessment using modified RECIST criteria and the characterization of the clinical-demographic landscape of this population and toxicity profile.

Results

The cohort consisted of 15 patients (median age: 65 years, range: 40-76), comprising eight males (54%) and seven females (46%). The median number of administered treatment cycles was seven for nivolumab (range: 2-26) and three for ipilimumab (range: 1-10). At the three-month evaluation, nine patients (60%) achieved a partial response, maintaining a stable response through the 12-month follow-up. Six patients (40%) experienced disease progression after the initial assessment, experiencing an OS of less than nine months. At the 12-month endpoint, the one-year OS rate was 60%, with nine patients alive and continuing immunotherapy. Treatment-related adverse events (TRAEs) occurred in 12 patients (80%), with Grade 3 or 4 TRAEs reported in four patients (26%). The most common all-grade TRAEs were diarrhea (26%), hypothyroidism/thyroiditis (20%), and rash (13%). Grade 3/4 toxicities included hepatitis (6%) and severe diarrhea (13%).

Conclusion

First-line treatment with combined nivolumab and ipilimumab demonstrates promising clinical efficacy, durable responses, and a manageable safety profile in Indian patients presenting with inoperable epithelioid MPM. These real-world outcomes align closely with global clinical trial benchmarks, supporting a chemotherapy-free dual-immunotherapy strategy. Larger prospective randomized trials are needed to validate these findings within the regional population.

## Introduction

Malignant pleural mesothelioma (MPM) is an uncommon but exceptionally aggressive malignancy arising from the mesothelial linings of the pleural cavity, strongly associated with historical asbestos exposure. The global disease burden carries a dismal prognosis, with a median survival of approximately one year post-diagnosis and a five-year survival rate hovering below 10% [1]. In India, the recorded incidence is estimated at approximately one in a million cases per year, though underreporting and diagnostic challenges often mask the true clinical volume [2].

For nearly two decades, systemic frontline therapeutic choices remained constrained to platinum-plus-pemetrexed cytotoxic chemotherapy regimens. While these combinations offer modest palliative benefits, they fail to deliver sustained long-term survival or durable disease control.

The therapeutic landscape shifted fundamentally following the landmark phase III CheckMate 743 trial, which demonstrated a statistically significant and durable overall survival (OS) advantage using first-line dual checkpoint inhibition with nivolumab (anti-PD-1 antibody) and ipilimumab (anti-CTLA-4 antibody) compared to standard of care chemotherapy [3]. This regimen effectively blocks synergistic coinhibitory pathways, restoring T-cell-mediated anti-tumor immunity. While globally adopted as a standard first-line choice, real-world clinical data evaluating this immunotherapy combination within the Indian demographic remains very scarce. The primary objective of this study was to evaluate the real-world OS, including median OS and one-year OS, and safety profile. This study addresses the therapeutic efficacy, safety parameters, and demographic characteristics of Indian patients undergoing frontline treatment with nivolumab and ipilimumab for inoperable epithelioid MPM.

The abstract of this study was presented at ISMPOCON-2024 and was published as a conference proceeding abstract in the Indian Journal of Medical and Paediatric Oncology. The current manuscript represents an expanded full-length retrospective analysis detailing the finalized 12-month overall survival tracking, structured cross-trial discussion metrics, and institutional ethics review parameters [4].

## Materials and methods

This single-center retrospective study reviewed patient medical records at a tertiary medical college hospital in Udaipur, Rajasthan, India, across a time span of January 2021 to August 2023.

Inclusion and exclusion criteria

This retrospective study reviewed adult patients aged 18 years or older at the time of treatment initiation with a baseline Eastern Cooperative Oncology Group (ECOG) performance status score between 0 and 2 [5]. Eligible individuals had an advanced, metastatic, or inoperable epithelioid malignant pleural mesothelioma histologically diagnosed and confirmed using contrast-enhanced computed tomography (CECT) or fluorodeoxyglucose positron emission tomography-computed tomography (FDG PET-CT) scan. Patients were excluded if they presented with alternative histological subtypes, such as sarcomatoid or biphasic variations. Additional exclusion criteria included a prior history of receiving systemic chemotherapy or targeted therapy, as well as a history of active autoimmune conditions requiring systemic immunosuppression.

Objectives

The primary objective of this study was to evaluate the real-world OS, including median OS and one-year OS, and safety profile of first-line nivolumab plus ipilimumab in Indian patients with inoperable epithelioid malignant pleural mesothelioma. Secondary objectives included the assessment of objective response rate (ORR) at first reassessment using modified RECIST criteria and the characterization of the clinical-demographic landscape of this population and toxicity profile.

Methodology

Relevant data of the patients was retrieved from the institute's medical records. All enrolled patients received a standardized weight- and protocol-driven immunotherapy schedule: nivolumab: 240 mg administered via intravenous infusion over 30 minutes, repeated once every two weeks and ipilimumab: 1 mg/kg administered via intravenous infusion over 30 minutes, repeated once every six weeks. Therapeutic cycles proceeded sequentially until documented objective radiological disease progression or the development of unmanageable treatment-limiting toxicity.

Demographic data, physical parameters, pathological and radiological reports were collected and analysed. Baseline FDG PET-CT imaging was done before starting therapy. 

Clinical and response assessments

Response evaluation was done after three months of therapy using FDG PET-CT scan and was categorized utilizing the modified Response Evaluation Criteria in Solid Tumors in malignant pleural mesothelioma (mRECIST) [6]. On the basis of first reassessment at three months, further therapy was continued. Those patients who showed either stable disease (SD), partial response (PR) or complete response (CR) were continued on same treatment and were followed up until death. Survival analysis was done after 12 months of follow-up.

Safety profiles and treatment-related adverse events (TRAEs) were closely recorded at every treatment interval. Toxicities were graded according to the National Cancer Institute Common Terminology Criteria for Adverse Events (CTCAE v5.0) [7].

Statistical analysis

For statistical analysis, data were entered into a Microsoft Excel (Redmond, WA, USA) spreadsheet. Data has been summarized as median, mean and standard deviation for numerical variables and count and percentages for categorical variables.

Time-to-event data were analyzed using the Kaplan-Meier method to estimate the cumulative survival probability of the cohort over time [8]. The survival time was calculated from the date of diagnosis to the date of progression or death. Median survival time along with its 95% confidence interval (CI) was calculated to summarize the cohort's trajectory. All statistical analyses and survival curves were generated using SPSS Statistics for Windows, Version 25 (IBM Corp., Armonk, NY, USA).

## Results

Demographic distribution

This study evaluated 15 eligible patients. The median age at initial diagnosis was 65 years, with a distribution range between 40 and 76 years. Gender stratification showed eight male patients (53.3%) and seven female patients (46.7%). The precise sex distribution across age sub-brackets is outlined below (Table 1). In 40-49 years age group, four (26.7%) were male and 0 (0%) were female. In 50-59 years age group, one (6.7%) was male and two (13.3%) were female. In 60-69 years age group, four (26.7%) were male and two (13.3%) were female and in 70-79 years age group, 0 (0%) were male and two (13.3%) were female. Out of 15 patients, eight (53.3%) patients were smokers and seven (46.7%) were non-smokers. Twelve (80%) patients belonged to rural areas while three (20%) belonged to urban areas. Seven (46.7%) presented with ECOG performance status 0, six (40%) presented with performance status 1, and two (13.3%) presented with performance status 2. History of asbestos exposure was not present in any patient. Baseline characteristics are outlined in Table 2.

**Table 1:** Sex distribution across age sub-brackets

Age (Years)	Male n(%)	Female n(%)	Total n(%)
40-49	4 (26.7%)	0 (0%)	4 (26.7%)
50-59	1 (6.7%)	2 (13.3%)	3 (20%)
60-69	4 (26.7%)	2 (13.3%)	6 (40%)
70-79	0 (0%)	2 (13.3%)	2 (13.3%)

**Table 2:** Baseline Characteristics ECOG: Eastern Cooperative Oncology Group [5], PS: performance status

Baseline Characteristics	
Median Age, years (range)	65 (40-76)
Sex, n(%)	
Male	8 (53.3%)
Female	7 (46.7%)
Smoking status, n(%)	
Smoker	8 (53.3%)
Non-smoker	7 (46.7%)
Area,n(%)	
Rural	12 (80%)
Urban	3 (20%)
ECOG PS, n(%)	
0	7 (46.7%)
1	6 (40%)
2	2 (13.3%)

Treatment exposure

The analysis revealed a median of seven completed cycles of nivolumab (range: two to 26 cycles) and a median of three completed cycles of ipilimumab (range: one to 10 cycles).

Efficacy and survival outcomes

**Figure 1:**
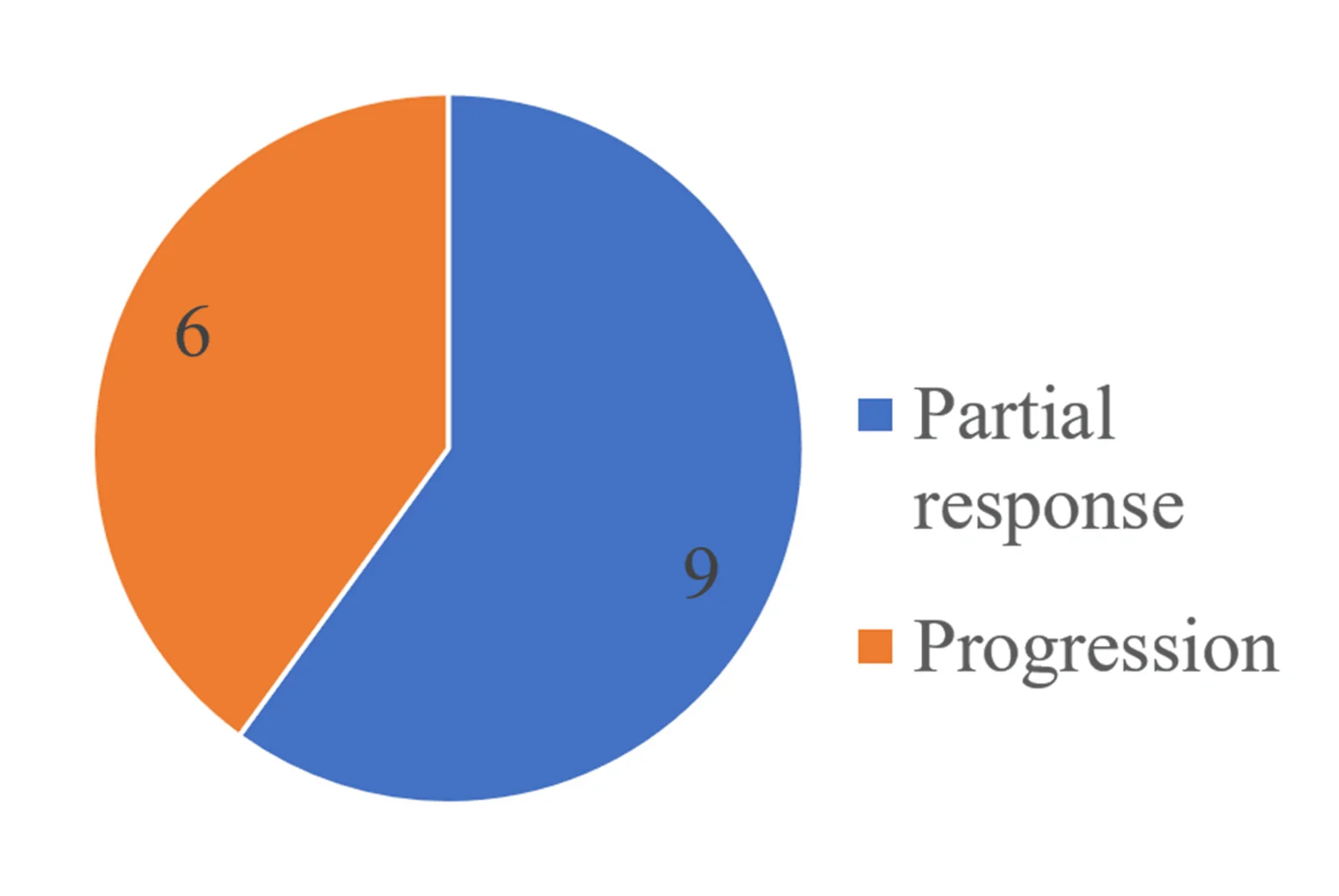
Response at three months

Radiological assessment at three months using FDG PET-CT revealed that nine patients (60%) achieved PR. This sub-cohort maintained clinical stability and persistent tumor control throughout the 12-month observation window. Conversely, six patients (40%) experienced early therapeutic failure or disease progression at or prior to the initial three-month scan. None of the patients achieved CR or SD. Thus ORR calculated was 60%. Figure 1 shows response on first reassessment at three months. All six progressing individuals exhibited rapid clinical decline, with survival failing to cross nine months despite subsequent salvage line chemotherapy.

During the follow-up, six events occurred, resulting in a cumulative event rate of 40%. The median overall survival time (95% CI) was not reached within the 12-month study period, as the survival probability remained above 50%. The estimated survival probabilities with 95% CIs at key time points were: 87% at three months, 80% at six months, and 60% at 12 months.

**Figure 2:**
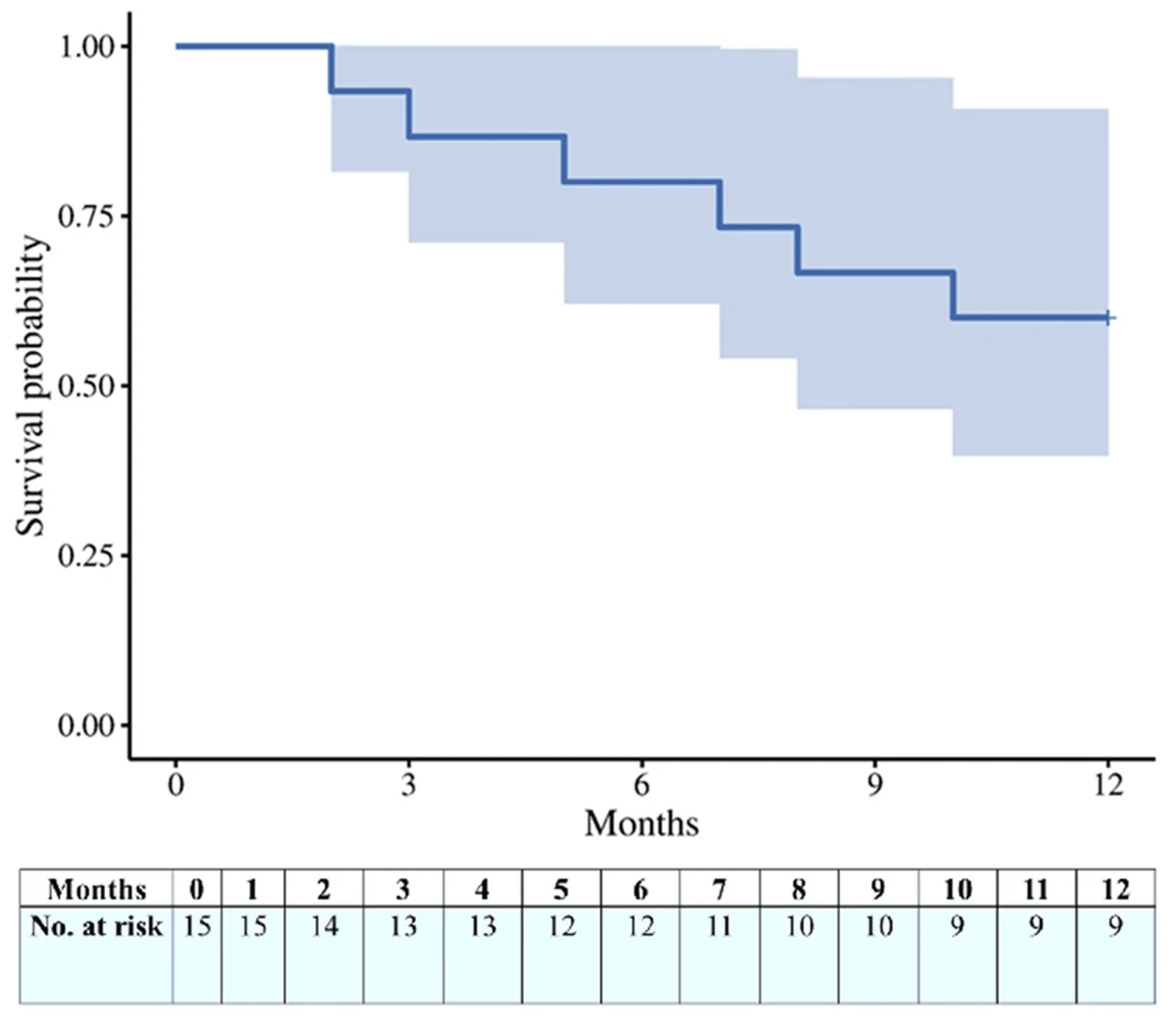
Kaplan-Meier curve of survival

At the end of the 12 months, nine (60%) patients remained alive and actively maintained on immunotherapy maintenance. The one-year OS rate for the entire study population was 60%. Figure 2 shows the Kaplan-Meier curve of survival.

Safety and adverse events profile

Immunotherapy-related toxicities of any grade manifested in 12 patients (80%). Severe Grade 3 or 4 TRAEs occurred in four individuals (26.7%). Gastrointestinal toxicities were the most frequent complication, with all-grade diarrhea recorded in four patients (26.7%), two of whom (13.3%) escalated to Grade 3/4 status requiring systemic corticosteroid intervention. Endocrine dysregulation was prominent, with three patients (20%) developing low-grade hypothyroidism/thyroiditis requiring regular levothyroxine supplementation. Cutaneous toxicity presenting as low-grade maculopapular rash occurred in two patients (13.3%). Grade 3/4 immune-mediated hepatitis occurred in one patient (6.7%), which responded to treatment suspension and steroid management. No treatment-related deaths were encountered. No new safety signals were observed. Treatment discontinuation was due to disease progression only; no toxicity-related discontinuation was seen. The profile of TRAEs is shown in Table 3.

**Table 3:** Adverse events profile.

Adverse Event Type	All-Grade Incidence, n (%)	Grade 3/4 Incidence, n (%)
Diarrhea	4 (26%)	2 (13%)
Hypothyroidism / Thyroiditis	3 (20%)	0 (0%)
Skin Rash	2 (13%)	0 (0%)
Immune-mediated Hepatitis	1 (6%)	1 (6%)
Any Adverse Event	12 (80%)	4 (26%)

## Discussion

This retrospective analysis provides crucial clinical insights into the real-world performance of first-line dual checkpoint inhibition within an Indian clinical setting. Historically, diagnosing and managing MPM in India has been a challenge characterized by extreme rarity and low institutional tracking. Early literature reviews by Rao (2009) emphasized that Indian centers rarely encountered MPM, capturing a minimal number of cases over multi-decade tracking periods, heavily plagued by an absence of clear asbestos exposure history and highly delayed, advanced-stage presentations [1]. Our modern cohort reflects a transitioning clinical landscape where superior diagnostic imaging helps identify these advanced cases, highlighting the critical need for effective frontline interventions.

Our observed one-year overall survival rate of 60% aligns remarkably well with the 68% rate observed in the definitive CheckMate 743 trial [3]. While our survival rate is slightly lower - likely due to late-stage regional referrals and a wider performance status allowance (ECOG 0-2) - our ORR (60% PR) was notably higher than the 40% reported in the trial. This variance is driven by our strict inclusion of the epithelioid histotype, which tends to respond more uniformly to initial treatment interventions.

When evaluating long-term trends through successive trial readouts, the three-year update of CheckMate 743 (Peters et al., 2022) confirmed a sustained, clinically meaningful survival benefit with dual checkpoint inhibition over standard chemotherapy, establishing a median overall survival of 18.1 months and showcasing that survival benefits were maintained over time regardless of histology [9]. More recently, the five-year update (Scherpereel et al., 2025) provided the definitive long-term validation of this regimen, revealing a clear survival tail/plateau where a clinically significant proportion of patients achieved long-term survival that was historically impossible with cytotoxic doublets [10].

In our study, 60% of patients maintained a stable response through 12 months, mirroring this international trend of sustained immunological control. However, our 40% early progression rate highlights a clear subset of non-responders who suffer rapid clinical decline, emphasizing the global medical mandate to uncover predictive biomarkers. Recent studies by Mitra S et al. (2025) suggest that evaluating liquid biopsies, specifically monitoring soluble mesothelin-related peptides (SMRP), may serve as an effective prognostic tool to identify these non-responding individuals prior to the initiation of frontline dual immunotherapy and futures researches are required to prove its efficacy [11]. 

From a safety and compliance perspective, our toxicological profile bridges seamlessly with international real-world datasets. The multicenter German MesoNet study evaluated 135 unselected patients and noted a one-year survival profile of 56.2% and an ORR of 34%. These real-world data points indicate that unselected, everyday clinical cohorts show slightly lower survival statistics compared to strict randomized trials, a trend that directly contextualizes and validates our institutional outcomes. Furthermore, the MesoNet cohort noted that severe Grade 3/4 toxicity occurred in 21.4% of patients receiving the regimen [12].

This baseline is reinforced by the Australian RIOMeso registry, which evaluated 98 unselected real-world mesothelioma patients and reported a one-year survival rate of 58% alongside a Grade 3/4 toxicity profile of 23% [13]. Both registries highlight that while combination immunotherapy can be highly toxic in an unselected real-world population compared to controlled trials, careful patient selection and proactive management can mitigate risks. Our institutional Grade 3/4 toxicity rate of 26% matches these international registry limits. Much like the observations in the MesoNet and RIOMeso data, these real-world records confirm that immune-mediated toxicities, such as severe diarrhea and hepatitis, remain highly manageable through standardized corticosteroid algorithms without escalating baseline mortality risks [12,13].

Our study shows a 60% one-year overall survival rate for epithelioid malignant pleural mesothelioma, which is consistent with the 66.4% survival rate reported in the long-term, real-world Meso-Immune (GFPC 04-2021) study. While our study reported a lower 26% grade 3/4 toxicity rate compared to the 35.6% observed in the French Meso-Immune registry, both studies demonstrate that dual immunotherapy is a manageable, viable treatment option outside of controlled trials [14,15].

The primary limitations of this study include its retrospective design, absence of a comparator group, potential selection bias, limited follow-up single-center framework, and modest sample size. Nonetheless, it provides pivotal validation that the survival benefits and toxicological boundaries established in pristine Western clinical trials translate safely into real-world Indian oncological practice. Toxicity rates were found similar across larger trials, however.

## Conclusions

In conclusion, this study demonstrates that first-line systemic administration of combined nivolumab and ipilimumab is a safe and good viable chemotherapy-sparing regimen option for patients presenting with metastatic or inoperable epithelioid MPM. The regimen achieved substantial objective structural responses and encouraging one-year overall survival outcomes that closely align with established global benchmark data, such as the landmark CheckMate 743 trial. Furthermore, the safety profile underscores the feasibility of this dual immunotherapy approach, as observed immune-mediated adverse events (imAEs) were entirely manageable through standard, guideline-driven immunosuppressive protocols without compromising therapeutic continuity.

These real-world findings complement the findings from randomized clinical trials and suggest transitioning away from traditional platinum-based doublets toward frontline dual immune checkpoint inhibition in patients with epithelioid histology. However, to definitively establish these outcomes within diverse demographics, large-scale, multi-center prospective randomized controlled trials are urgently warranted across the Indian subcontinent. Future research must focus on identifying predictive biomarkers to refine patient selection, exploring cost-effective dosing strategies to optimize economic delivery in resource-constrained settings, and capturing mature, long-term survival data to confirm the durability of this therapeutic approach.
